# Eggs in Rectal Feeding

**Published:** 1892-08

**Authors:** 


EGGS IN RECTAL FEEDING.—Dr. A. Huber (*Le Bull. Med.*) has used eggs, to which a gramme (fifteen grains) of sea salt has been added, with success in rectal feeding, without ever observing any albuminuria. Too much salt is liable to cause profuse diarrhœa. The injection should be carried well up into the intestines, by means of a soft rubber tube.—*Ex.*

